# Midwifery

**Published:** 1838-04

**Authors:** 


### MIDWIFERY.

### *Report of the Berlin Lying-in Hospital, from October* 1, 1829, *to December* 31, 1835. By Professor Busch.

[The original Report, of which the following is a condensed analysis, occupies nearly two whole Numbers of the valuable Journal in which it appears.]

This institution is attached to the university, being expressly intended by the Prussian government for clinical instruction; and has recently, it appears, been erected in a more convenient part of the town, being close behind the building of the university itself. The patients are divided into two classes,—those who are delivered in the hospital, and those who are attended at their own houses. Prof. Busch is the successor of the celebrated Elias von Siebold, and has carefully recorded everything which has been worthy of interest, both in a statistical as well as practical point of view. We have only to complain that so much time has been allowed to elapse before publishing a Report, which ought, at the longest, to have been every two years. The quantity of matter collected would not have been so bulky. In the present instance it is much too unwieldy for insertion in a Journal, and would have appeared infinitely better in a separate work, like that of Dr. Collins.

The following is a condensed summary of the general view which Prof. Busch has given of his practice for the above period, aud our readers will derive interest in comparing the results with those of Dr. Collins, as given in our Third Number.

| | | | |
|---|---|---|---|
| Total number of labours | 2056 | Boys | 1061 |
| Single births | 2035 | Girls | 1000 |
| Twin births | 21 | Sex not ascertained | 16 |
| Total number of children and fœtuses, | 2077 | Women delivered alive | 2054 |
| Mature and premature children | 45 | after death | 2 |
| Abortions | 32 | Primiparæ | 992 |
| Multiparæ | 1064 | Forceps cases | 178 |
| Patients recovered | 2016 | Extraction | 55 |
| Patients died | 38 | Turning effected by altering the patient's position | 4 |
| Children born alive | 1913 | | |
| dead | 132 | Turning with the head foremost | 4 |
| Children which died during the first | | nates foremost | 2 |
| three weeks | 92 | feet foremost | 57 |
| Presentations of the head | 1911 | Accouchement *forcé* | 5 |
| face | 18 | Artificial premature delivery | 3 |
| nates | 47 | Perforation | 6 |
| knees | 2 | Embryotomy | 2 |
| feet | 28 | Cæsarean operation during life | 1 |
| Total number of unnatural presentations | 54 | after death | 2 |
| Presentations of the shoulder | 43 | Reposition of the prolapsed cord | 3 |
| back, breast, sides, &c. | 11 | Artificial extraction of the placenta | 47 |
| Presentation of the arm and head | 86 | Incision of the perineum | 3 |
| Labours without assistance | 1711 | | |

Among the remarkable phenomena occurring *during* pregnancy, we may notice that of menstruation. Prof. Busch observes that, in several cases, menstruation appeared once, twice, and even three times, after conception. The patients had all borne children before, and the circumstance appeared to have no peculiar effect upon the pregnancy. Two remarkable cases, however, occurred, where the catamenia did not appear *except during pregnancy.* The first was in the woman's second pregnancy: she stated that she had never menstruated until her first pregnancy, when the catamenia appeared and returned regularly during the whole time. She was delivered of a dead child in the eighth month, after which the catamenia ceased until she again became pregnant, two years afterwards: it now returned at regular intervals. When advanced four months, she applied at the hospital, fearing that the continuance of the discharge would have an injurious effect upon the life of the child. In spite of bleeding just before the expected recurrence of the periods, horizontal posture, &c., it continued to appear until the seventh month, when it stopped. Labour came on a few weeks before the time, and she was delivered with ease of a small living child.

*Retroversion.* A well-marked case of this displacement is given, which occurred in a multipara in the fourth month, after lifting a heavy weight. Violent pain of the loins, with obstinate constipation and retention of urine, followed. The distended bladder reached above the navel, the os uteri was high up behind the symphisis pubis, the fundus deep in the hollow of the sacrum. Six pints of urine were drawn off, and the uterus was easily restored to its natural position: abortion followed ten days afterwards. Prof. Busch states that, according to his large experience, evacuation of the bladder is usually sufficient, and that, where the uterus will not return, if an elastic catheter be left in the bladder for a short time, the uterus will either return spontaneously, or at any rate admit of being easily reduced. This by no means agrees with the experience of the English and American practitioners, who have shewn, in a great number of cases, that the uterus may be so impacted in the pelvis as to be incapable of reposition, although the bladder be regularly and completely evacuated.

*Protracted Labour* occurred in several instances: in upwards of thirty cases it lasted three days, and mostly without injury to the mother and child. Where no peculiar indication existed for the interference of medical or operative assistance, the patient was recommended to keep quiet, and the case was left to the natural powers.

*Weak Labour-pains.* The unripe Secale cornutum, which had not been kept above a year, was given in 175 cases of this sort, but never until the os uteri was fully dilated. The efficiency of this remedy was so distinctly marked, that in by far the majority of cases the labour was terminated without assistance: in 115 cases its action was effective and speedy; the aid of the forceps was required in 27, of turning in 6, and in 7 cases the body required extraction after the head had been expelled; in twenty cases it appeared to have no effect. In several cases of inertia uteri after delivery of the child, with retention of the placenta or hemorrhage, it was given with good effect. It was always given in the form of powder, in doses of ten grains, repeated every ten or fifteen minutes. In the 175 cases where it was given, 177 children were born; 142 were born alive, 18 in a state of asphyxia (from which they recovered), and 17 were born dead; of which 7 had evidently been dead some time previously, and of the 10 others, which died during labour, 2 lost their life by turning, 1 from presentation of the nates, 2 from prolapsus of the cord, 1 from contracted pelvis and long-continued impaction of the head in it, 1 from the protracted dilatation of the external parts, 1 from a peculiar deformity of all the extremities, and 1 from no very peculiar cause; so that, out of 177 children, only one death occurred which could be possibly attributed to the use of the ergot.

*Convulsions.* Eleven cases occurred: five were of the mild form, and six of the genuine and mostly fatal eclampsia: all were primiparæ. Four died.

*Varicose Veins of the Labia and Vagina* were observed in several cases, in various degrees: in some the swelling was as big as a large fist, but labour passed over without any hinderance, except in two cases where the tumour burst. In one no hemorrhage took place, but the blood infiltrated itself into the cellular tissue of the left labium, and distended it immensely: an incision was made to discharge the collected blood and allow the head to pass, and the wound healed in fourteen days. In the other case, the varix was on the anterior wall of the vagina, and burst, producing a smart hemorrhage: this was restrained by a cloth dipped in vinegar, and the labour terminated favorably.

*Placenta prævia* occurred in thirteen cases, varying considerably in degree, and requiring different modes of treatment. Two cases proved fatal to the mother, where there had been great delay in sending for assistance. In eight cases the child presented with the head, and in five with the shoulder.

*Hemorrhage* followed the birth of the child in 124 cases, only two of which proved fatal; and in these assistance had not been sent for until they had become quite hopeless. In speaking of those hemorrhages which arose from inertia uteri, where the placenta is still in the uterus, Prof. Busch recommends introducing the hand for its removal. The practice is very general in this country, but has been very little adopted upon the continent; and the reasons which he assigns are precisely those which have been given by the English authors. Prof. B. strongly opposes any officious interference in the expulsion of the after-birth: with this we cordially agree; but must differ from him most completely where he recommends it to be left entirely to the natural means of expulsion *for the first few hours,* when no unusually firm adhesions or other abnormal circumstances exist.

*Deformity from Rickets.* Prof. B.'s experience tends to confirm the fact that considerable distortion of the spine or thorax is rarely accompanied with deformed pelvis.

Five cases of extreme spinal deformity occurred: all were delivered of living children, and without any assistance, except the last, where the forceps was required. This was an extraordinary case of deformity: besides an extreme degree of spinal distortion from rickets in early life, the upper and lower extremities were so completely crippled and paralysed that the patient could neither walk nor stand, but was only able to lie down or sit. The pelvis was not very spacious, and so greatly inclined that the axis of it was horizontal; the child nevertheless presented with the head. It was no easy matter to apply the forceps, on account of the degree of pelvic inclination, the outlet being directed quite backwards: the operation, however, succeeded, and a living child was extracted. Three cases of an unusual species of deformed pelvis were observed, viz. the *pelvis æqualiter justo minor*, or pelvis equally too small in every direction: it has been scarcely, if at all, noticed in this country, and certainly is not of very frequent occurrence to any considerable extent. The first case was a presentation of the breech: the head was delivered by the forceps; the child was dead; the pelvis measured half an inch too small in every direction. In the second case, which was a head presentation, the delivery was effected by the forceps, but not without the greatest efforts: the child was stillborn, and the mother died in a day or two after from peritoneal inflammation. The third case required perforation: this also terminated fatally, the forceps having been previously applied, and considerable efforts made without success. On examination after death, every diameter of the pelvis was three-quarters of an inch smaller than usual: in appearance it resembled that of a child.

*Artificial Premature Labour* was induced in only three cases: we will briefly state the particulars of one. The conjugate diameter was only three and a quarter inches: it was determined to open the os uteri by the dilator. The bowels having been well opened on the preceding day, and the abdomen rubbed with some warm almond-oil, some infus. chamæm. and oil were thrown into the vagina, and the dilator, properly warmed and oiled, introduced about three-quarters of an inch into the canal of the cervix. Five gentle efforts at dilatation were made, producing no pain, but merely a sense of tension: this was repeated noon and evening, premising each time the injection; and she was put upon an antiphlogistic diet. The following day, the mucous secretion from the vagina had increased; its parietes were warm and more cushiony: the dilating efforts were repeated three times, and to a greater extent. On the third day, the os uteri was open about the size of a shilling; the pains were tolerably good, but went off towards night, and the dilator was used again on the following day. Slight febrile symptoms appeared on the fifth day, and the dilator was intermitted until the sixth. The pains returned during the following night; the os uteri became fully dilated, the vagina was relaxed and soft, the bladder of membranes distended; but, as no presenting part could be felt, a faulty presentation of the child was surmised. It was determined to bring down the head, if possible: the patient was therefore placed upon her back, the right hand passed into the vagina, the membranes ruptured, and the head, which was resting against the left ilium, was guided into the brim of the pelvis. An immense quantity of liquor amnii was discharged; the head entered the brim in the first position; active pains quickly followed, and a living child was born in about a quarter of an hour after the rupture of the membranes. It weighed only two and a quarter pounds. The mother did well; but no mention is made of the child, which, from its diminutive size, in all probability died.

*Anchylosis in the Extremities of a Fœtus* occurred as a very unusual obstruction to labour. The head was delivered by the forceps, but the body would not follow. As no cause of obstruction could be discovered, a gentle, and then more powerful, traction was used: this was followed by a cracking sound, and the upper part of the trunk passed through the os externum: here, again, it stopped, but still, as no cause of obstruction could be discovered, and as the child was dead, another traction was made, with a repetition of the cracking sound, and the child was delivered. On examination, it was found that all the joints of the extremities were anchylosed in the usual position of the fœtus in utero; so that the ossa humeri, and then the ossa femoris, had given way. The child had been dead some time.

*Vagitus Uterinus.* A well-marked case of this singular phenomenon occurred in a shoulder presentation. The child was afterwards turned, and delivered in a state of asphyxia, from which it recovered. Professor Busch observes, that he has met with several cases of this sort, where the shoulder had presented and the liquor amnii escaped.

*The Cord round the Child's Neck,* in two cases, was drawn so tightly that a deep groove was produced, and yet without injury to the child's life. He very properly directs that the cord should be passed over the shoulders.

*Length of the Cord.* The longest cord measured forty-six, the shortest six, inches.

Insertion of the cord into the membranes was observed in ten cases. In four, the point of insertion was at the opposite side of the ovum to where the placenta was situated. In one case, there were three arteries and one vein which entered at opposite sides of the ovum. In an experience of thirty years, Professor B. has never observed any injury of the vessels in these cases where the membranes were ruptured.

*Turning with the Feet foremost* was performed in forty-four cases, with the highly favorable result that only three children lost their lives from the effects of the operation. Professor Busch ascribes this unusually small mortality among the children thus delivered to the circumstance of his instantly applying the forceps the moment the head experienced any hinderance when passing through the pelvis. The fact is interesting; and certainly the proportion of deaths is by far the smallest we have known of. In cases where turning was unusually difficult, he invariably premised a dose of opium, threw up an oleaginous injection into the vagina, and put the patient on her left side. He has given an interesting case where the shoulder presented in the same patient in six successive labours; but no attempt was made to ascertain the cause on which this peculiarity depended.

In giving directions as to the position in which the patient should be placed for turning, he says "the patient should be placed on the same side as the hand which the practitioner is about to use for turning the child." It is on the same principle that many accoucheurs in this country find it more convenient to use the left hand in turning, the patient being mostly placed upon the left side; and there is no doubt that, where the feet are high up and forwards, we can thus reach them more easily.

*Central Laceration of the Perineum* occurred in a case where the delivery was effected by the forceps. As the head was passing, the perineum was felt by the supporting hand to tear slowly asunder, in spite of the greatest care, leaving the frænulum uninjured, forming, as it were, a bridge across the anterior portion of the opening. The patient was kept upon her side; Goulard lotion was constantly applied, and the opening contracted so rapidly, that on the third day it would barely admit the finger, and in four weeks it was quite cicatrized. We have ourselves observed a similar case, where the child passed *through* the perineum, but where a minute orifice remained, which did not give way and dilate in a subsequent confinement. We cannot, however, quite agree with the Professor that this is the most favorable species of perineal laceration: the prognosis must, of course, depend upon the extent of the laceration, and especially whether it extends into the rectum or not.

*Perforation* was used in six cases, and under circumstances which we cannot but deprecate. The forceps had been previously applied, and without success, in each case: to the injuries thus produced must the deaths of two patients be in a great measure attributed.

*The Cæsarean Operation* was performed three times: once on a living woman, on account of deformed pelvis: the patient died, but the child was preserved. The two other cases were after sudden death, in neither of which was the child saved.

*Puerperal Fever* shewed itself in thirty cases, although not appearing in an epidemic form. The prevailing character of the disease was inflammatory, requiring local and general abstraction of blood and the exhibition of calomel and antimony. Of these thirty cases, seven proved fatal: extensive effusions of seropurulent fluid were found in the abdominal cavity. In one case only were there any marks of inflammation and purulent effusion in the veins. In every case the disease first shewed itself between the second and fourth day.

*Phlegmasia Dolens* occurred in five cases, of which one proved fatal: with the exception of this last, the disease did not appear till the seventh day. In every case the pain was very intense; the swelling commenced in the upper part of the thigh, and spread rapidly either over the whole extremity, or, at least, as far as the leg, extending also sometimes to the groins. Professor B. considers that these five cases belong to what he calls inflammation of the cellular tissue, no inflammation of the veins having been observed.

We subjoin a list of the fatal cases:

| | |
|---|---|
| Hæmorrhage from placenta prævia | 2 |
| after birth of the child | 2 |
| Exhaustion after turning, which had been delayed | 1 |
| Hæmorrhage in the puerperal state | 1 |
| Metritis and gangrene | 3 |
| Putrescence of the uterus and phlebitis | 4 |
| Phlegmasia dolens | 1 |
| Eclampsia in partu | 4 |
| After the Cæsarean operation | 1 |
| Puerperal fever | 7 |
| Typhoid fever | 3 |
| Abdominal typhoid fever | 2 |
| Apoplexy | 1 |
| Mania apoplectica | 1 |
| Gangrene of the lungs after pneumonia | 1 |
| Phthisis | 4 |
| Total | 38 |

*Neue Zeitschrift für Geburtskunde.* Vol. xv. 1837.

*Account of a Case of Ruptured Uterus.* By Dr. NAEGELÉ, Jun.

[THE following case, from the extraordinary nature of the circumstances connected with it, we believe to be quite unprecedented.]

A healthy peasant woman, æt. 35, of middle size, and delicately formed, mother of four children, and in the latter half of her pregnancy, (she reckoned that she had still six weeks to go,) received a violent blow on the abdomen from the pole of a waggon. She felt at the moment a severe tearing pain, and was thrown down with considerable force, but managed to creep a little way from the waggon, where her husband found her, and conveyed her home to bed in a fainting state. On coming a little to herself, she complained of a constant bearing-down pain in the abdomen, which prevented sleep during the night; but she had no return of the fainting. Besides the above symptoms, Dr. N. found her with a flushed face; pulse ninety, full and sharp; the bowels had been only once moved after several injections; she could pass water, but it produced pain and scalding. The abdomen was slightly tympanitic, but not tense. Between the umbilicus and pubes, somewhat to the left, a round circumscribed tumour was to be felt, rising in the middle to the height of an inch and a half, but flattened off towards the sides, and about six inches in diameter. The parietes were unchanged, except that there was a small circular bruise. Beneath the integuments covering this tumour, the nates and foot of a fœtus could be felt with the greatest distinctness, over which they were easily moved. She complained of pain on motion or on pressing the tumour. On pressing his hand deeper into the abdominal parietes above the navel, Dr. N. fancied he could feel the fundus uteri. Examination per vaginam presented nothing unusual. The head presented naturally, and neither blood nor water had come away from the vagina; she had no pains, nor were there any signs of incipient labour. The movements of the child, which had been very brisk before the accident, had ceased immediately after; and this was moreover confirmed by auscultation. The alarming symptoms which are usually observed in cases of ruptured uterus were entirely absent: nevertheless, the nature of the accident, the peculiar character of the above-mentioned swelling, and the distinctness with which the parts of the child could be felt immediately beneath the abdominal parietes, were strongly in proof of the uterus having been ruptured.

Under the existing circumstances, Dr. Naegelé considered that neither gastrotomy nor artificial delivery *per vias naturales* were indicated; as, in the first case, the child was evidently dead, and, in the second, there was not even a trace of commencing labour: he therefore contented himself with bleeding her to fourteen ounces, on account of the pain of head and state of the pulse, enjoined strict quiet, and requested the midwife (who was the only medical attendant in the village,) to send for him as soon as labour made its appearance. On the following day, the report was that no change had taken place, except that a cough, to which she had been many years subject, had increased, and aggravated the pain of the abdomen. Some dec. alth. and ext. hyosc. were ordered, and several days passed without hearing of her. "On the 5th of July," says Dr. N., "a messenger at length arrived at midnight, with the information that pains had come on the day before, in the forenoon, and that she had been partly delivered of a putrid fœtus in the afternoon; but, as it was not entirely expelled, my assistance was required. I arrived at four in the morning, and learned from the midwife that the os uteri had gradually dilated, the head had descended lower into the pelvis, and had at length been expelled; but, strange to say, no bladder of membranes had formed, nor had a drop of liquor amnii come away during the labour; that, when the fœtus was delivered as far as the breast, it had there stuck, and, on her pulling at the body, it had given way in the vicinity of the lumbar vertebræ, leaving the rest behind. The placenta had passed of itself, without any serious discharge. On examining the portion of the child which had been expelled, and which was in a high state of putrefaction, I found everything as she had described. The unfortunate patient lay without motion in her bed, with well-marked signs of metritis: the face betrayed the peculiar and painful expression of suffering which is so constantly observed in severe uterine disease; the anxious countenance; confusion of mind; the pulse from 110 to 120, somewhat hard and tense; the skin dry and rough; the temperature of the abdomen increased, but the extremities cool; tongue dry, thirst and nausea; the bowels had been relieved by an enema, and she had passed water; the breasts were flaccid, and there were no traces of lochia. The abdomen was more distended than before, but the tumour itself had diminished; the parts of the child were less evident, but the nates and a foot could still be distinguished. The slightest touch produced insupportable suffering." On passing his whole hand into the vagina, the temperature of which was much increased, Dr. N. found the os uteri externum very high up and directed backwards: it was sufficiently open to admit two fingers. A leg of the child projected into the vagina, round which, at the knee-joint, the os uteri internum was firmly contracted. Repeated attempts were made to extract the foot, without success; for, besides the firmness with which the os uteri was contracted, the putrid state of the extremity prevented any firm hold being applied, and only produced the severest sufferings. In the hopes of relaxing the os uteri, she was bled to sixteen ounces, but without effect: the leg was therefore removed at the knee, warm fomentations were applied to the abdomen, strict quiet enjoined, with general antiphlogistic treatment and attention to the bowels.

No further news of the patient was received until the 10th of July, when Dr. N. was informed that she had been in severe suffering for two days previously; that the tumour had inflamed, and burst on the 8th; that a foot had protruded, at which the midwife had pulled until the whole remaining portion had been removed, and that the opening was very large.

Dr. N. saw her again on the 14th. The portion of the child which had been expelled from the abdominal tumour was examined, and was found to consist of the pelvis, with the entire lower extremity of one side and the thigh of the other. "To our astonishment," says Dr. Naegelé, "we found the patient, who, at our last visit, we thought could scarcely survive twenty-four hours, in a very comfortable state. There was but little fever, the pulse was about ninety, thirst moderate, appetite tolerably good, strength improved; her only complaint was that she did not sleep; there was no trace of lochia, and but little milk in the breast. In the place of the abdominal tumour was a round circumscribed opening in the fleshy wall of the abdomen, of about five inches in diameter, from which a quantity of mucus was discharged, and about an inch and a half in depth. On the right, the whole thickness of the abdominal wall was as if it had been cut through with a knife, beneath which the finger could be introduced for some extent: on the left side of the opening was a red globular mass, which had united with the abdominal integuments, and which appeared to be the uterus, as it rose distinctly when the lower portion of the uterus was pressed upwards by the finger per vaginam. Inferiorly, the finger was stopped by the adhesions which had formed in all directions at the bottom of the abscess; on the right side, a portion of intestine was visible. The wound was covered with simple dressing, and above this with a poultice; bark was ordered, with a mild but nourishing diet, and directions were given for ensuring proper relief in the bowels.

On the 19th July, the opening had diminished to a third; the portion of intestine, as also the red coloured mass, had retracted somewhat; there was a free discharge of healthy pus; she was perfectly free from suffering, except that one of the inguinal glands had inflamed and suppurated, causing her a good deal of pain. The simple treatment, as above mentioned, was continued, and a poultice applied to the groin; a more nourishing diet was also directed to be used. The abscess burst in due time, the strength continued to improve, and, when he visited her on the 17th of August, Dr. N. found the opening of the abdomen entirely closed, leaving merely a small scar.

Her health and strength continued to improve, and the catamenia returned on the 12th of August. *Neue Zeitschrift für Geburtskunde.* Vol. v.

---

***Resuscitation of an Infant after Interment.*** By Dr. Wagner.

[The following curious case, which, we are assured by Dr. Wagner, rests on unquestionable authority, occurred last summer in Germany, and became the subject of a criminal investigation: it is interesting in many points of view, but especially in relation to medical jurisprudence. We content ourselves with subjoining to the case the pertinent but brief remarks given by the narrator.]

An unmarried servant-maid became pregnant, and, although herself aware of the fact, persisted in denying it, and took every pains to conceal it. At length she was seized with labour-pains, in the day-time, and was obliged to give up work. She complained of severe pain in the belly, which she attributed to cold, and persisted, even afterwards, that she so thought at the time, not believing the period of her delivery arrived. She slept in the same room with two other maid-servants; and, on the night in question, as she stated on her examination, the pains having increased, she got up, towards morning, in order, as she thought, to relieve her bowels. For this purpose she went to a wooden tub in the room, and, as she was sitting down, the child passed rapidly from her into the empty vessel. It was only then, she said, that she became aware of the nature of her pains. She did not closely examine the child, but was certain that it neither moved nor cried. The cord was, no doubt, torn at the time, and she made no attempt to tie it. Regarding the event as a miscarriage, she took up the tub with its contents, carried it to a sandpit about thirty paces distant, and threw the child into a hole in the sand, which she found already made. She covered the place up with sand and turf, and pressed the sand firmly in with the hand, in order, as she said, that the dogs might not reach the child. She returned to her bedroom, first calling up the man-servant at the stable door, and found her fellow-servants still sleeping. She awaked them; and feeling tired, sat down on a stool while they were getting up. One of the women, who had been awaked during the night by her groans, seeing some blood on the floor, and suspecting what had happened, asked her if she had not been confined, and had made away with her child. She replied "Do you take me for an old sow?" Having their doubts, however, the women followed the traces of blood, and came to the sand-pit, where they were struck by the appearance of the recently-disturbed ground. Fetching a spade, they discovered the child about a foot below the surface. On the access of the air, by the removal of the turf and sand, the child, still lying in the pit, began to cry, and was immediately taken up and carried to its mother, (from whom in the meantime the placenta had come away,) who washed it, and laid it on the bed. About an hour afterwards, the mother carried the child to her own mother in a neighbouring village; and then, for the first time, the navel string was tied. Another woman gave the infant the breast; and the mother, after being confined to bed for a few days, was delivered over to judicial examination. Her confession corresponded with the testimony of her witnesses, but she persisted in asserting that she considered the event as a miscarriage, and denied all intention of making away with the child. The investigation furnished no ground for disbelieving her assertion, although the child was proved, by medical testimony, to have been carried to the full term, and was in every respect healthy, except that it had a club-foot. From the evidence adduced, and the confession of the mother, the child must have remained at least a quarter of an hour under-ground; and, as it lay a foot below the surface, and as the sand and turf, according to the mother's confession, had been closely pressed upon it, no air could have readily reached it. It is hardly possible that the child could have breathed before interment; as, if the respiratory process had been once begun, the subsequent inhumation must have infallibly suffocated it. On the contrary, it is quite conceivable that the child was born asphyxiated, was buried in this state, and only then began to respire and assume independent vitality, when it was for the second time exposed to the air.

The above case is interesting in regard to the physiology of asphyxia, or apparent death. It is no less interesting in a medico-legal point of view, and in several respects. It has an important bearing on the doctrine of the hydrostatic test, and the objection made to it, that it indicates only the preexistence of life with respiration, not absolute life with or without respiration The possibility of such a condition, and even of longer duration, is proved by this case; and it hence becomes a question in how far such an event is to be admitted in judgment, since the existence of this kind of vitality can, in general, be neither proved nor disproved. It is also worthy of notice that the navel-string remained for such a length of time untied without hemorrhage. The subject is, moreover, interesting in a purely judicial point of view, inasmuch as no penal law bears on the case; and yet we cannot here regard the conduct of the accused as not meriting punishment. She cannot be punished for intended infanticide, since all the evidence tends to exculpate her from this charge: she cannot be punished for concealing the pregnancy and birth, although proved, because, according to the law (of Germany) punishment does not ensue if the child is living; and no mention is made of any penalty for the interment of a living but apparently dead-born child. *Medicinische Zeitung. January* 17, 1838.

---

*Observations on the Etiology of the Thrombus, or Bloody Tumour of the Head of the Fœtus in tedious Labours.* By Vicente José de Carvalho, m.d., of Oporto.

This is a paper of considerable length, and displaying much research into the subject mentioned. The writer describes, with much candour but some prolixity, his successive changes of opinion. He first entertained, and then rejected, the view which assimilates these tumours to sanguineous swellings the effect of contusion; his rejection of it being founded on the obvious fact that the tumour is formed, not on the part of the head exposed to pressure and bruising from the irregularity of the bones of the pelvis, but on the vertex corresponding to the orifice of the uterus, where there is little or no pressure. He then adopted the idea that the swelling arose from the impediment to the return of the venous blood from the dependent and unpressed part, where thrombus forms in consequence of the circumference of the head being tightly embraced by the neck of the uterus; the tumour being thus assimilated to the condition of the lower part of a limb, round the upper part of which a tight ligature is bound. Some observations made in the dissection of the head of a fœtus, on which such a tumour existed, led the author again to modify his opinion. The tumour being opened, and the interior of its base exposed, the occipito-bregnatic circumference of the head being bound firmly with a cord, dark blood was seen to ooze from within the cranium, through the veins which penetrated the parietal foramina situated at the base of the thrombus. On drawing the ligature more tightly, there began to flow from within the cranium vermiform coagula of some inches in length, proceeding from the superior longitudinal sinus; which, on subsequent examination, was found empty.

The following are the conclusions which Sr. V. J. de Carvalho thinks himself justified in deducing from this anatomical fact and all the phenomena of the tumour:

1. The thrombus, or sanguineous tumour of the head of the fœtus, ought to be considered as a true *diverticulum* of cerebral blood, in which a portion of this liquid is accumulated on each occasion that the head is delayed for a considerable period in the pelvis. 2. On this occasion the encephalon and the head of the fœtus diminish absolutely in volume, which will be less in proportion to the quantity of blood received in a short time into the *diverticulum.* 3. The blood contained in a thrombus should be considered as a cerebral bleeding, which nature performs in lingering labours, to prevent the apoplexies, effusions, and other accidents to which the fœtus would be liable on such occasions. 4. The cavity of a thrombus represents that of a vessel hermetically sealed, where the blood is preserved liquid in a temperature equal to that of the part whence it has proceeded. 5. Nature performs a true transfusion with the blood of the same individual, when she causes that which she had preserved for some time in the *diverticulum* to re-enter the circulation. 6. The thrombus communicating directly with the snperior longitudinal and lateral sinuses, the tumour should never be opened, on account of the risk of the introduction of air into these canals, which would be necessarily fatal. 7. The oblong figure which the thrombus presents occasionally, rendering more acute the pyramidal form which the head possesses in occipital presentations, contributes, in the manner of a wedge, to the dilatation of the neck of the uterus and of the vagina in the act of parturition. 8. As the compression of the body and head of the fœtus, conjointly with the dependent situation of this part, was the cause of the formation of the thrombus, so the cessation of the pressure on these parts and the altered position of the infant, assisted by gentle compression on the tumour, suffice for its dispersion in a few hours.

[The part of the author's paper which we think the most questionable is the assumption of the fluid condition of the blood within the tumour for a considerable period, and the reasoning founded upon it. This is certainly contrary to what is observed of blood when extravasated, even within the body.

The patriotic author concludes his paper by expressing the hope that this new etiology of thrombus, conjoined with other national discoveries, will tend to elevate Portuguese obstetricy above the contempt of certain foreigners of whom he complains.]

*Jornal de Sociedade das Sciencias Medicas de Lisboa, for Dec.* 1836.

